# The Enterics for Global Health (EFGH) *Shigella* Surveillance Study in The Gambia

**DOI:** 10.1093/ofid/ofae049

**Published:** 2024-03-25

**Authors:** Bakary Conteh, Henry Badji, Abdoulie F Jallow, Mehrab Karim, Alhagie Manneh, Belali Keita, Golam Sarwar, Bubacarr E Ceesay, Sheikh Jarju, Abdoulie M J Jabang, Ebrima Baldeh, Usman N Ikumapayi, Ousman Secka, Martin Antonio, Anna Roca, Umberto D’Alessandro, Karen L Kotloff, M Jahangir Hossain

**Affiliations:** Medical Research Council Unit The Gambia at the London School of Hygiene and Tropical Medicine, Fajara, The Gambia; Medical Research Council Unit The Gambia at the London School of Hygiene and Tropical Medicine, Fajara, The Gambia; Medical Research Council Unit The Gambia at the London School of Hygiene and Tropical Medicine, Fajara, The Gambia; Medical Research Council Unit The Gambia at the London School of Hygiene and Tropical Medicine, Fajara, The Gambia; Medical Research Council Unit The Gambia at the London School of Hygiene and Tropical Medicine, Fajara, The Gambia; Medical Research Council Unit The Gambia at the London School of Hygiene and Tropical Medicine, Fajara, The Gambia; Medical Research Council Unit The Gambia at the London School of Hygiene and Tropical Medicine, Fajara, The Gambia; Medical Research Council Unit The Gambia at the London School of Hygiene and Tropical Medicine, Fajara, The Gambia; Medical Research Council Unit The Gambia at the London School of Hygiene and Tropical Medicine, Fajara, The Gambia; Medical Research Council Unit The Gambia at the London School of Hygiene and Tropical Medicine, Fajara, The Gambia; Regional Health Directorate Upper River Region, Ministry of Health and Social Welfare, Basse, The Gambia; Medical Research Council Unit The Gambia at the London School of Hygiene and Tropical Medicine, Fajara, The Gambia; Medical Research Council Unit The Gambia at the London School of Hygiene and Tropical Medicine, Fajara, The Gambia; Medical Research Council Unit The Gambia at the London School of Hygiene and Tropical Medicine, Fajara, The Gambia; Medical Research Council Unit The Gambia at the London School of Hygiene and Tropical Medicine, Fajara, The Gambia; Medical Research Council Unit The Gambia at the London School of Hygiene and Tropical Medicine, Fajara, The Gambia; Center for Vaccine Development and Global Health, University of Maryland School of Medicine, Baltimore, Maryland, USA; Department of Pediatrics, University of Maryland School of Medicine, Baltimore, Maryland, USA; Medical Research Council Unit The Gambia at the London School of Hygiene and Tropical Medicine, Fajara, The Gambia

**Keywords:** diarrhea, Gambia, healthcare, prevalence, *Shigella*

## Abstract

**Background:**

The Gambia, located in West Africa, is one of 7 country sites conducting the Enterics for Global Health (EFGH) *Shigella* Surveillance Study to establish incidence and consequence of *Shigella*-associated medically attended diarrhea among children 6–35 months old.

**Methods:**

Here we describe the study site and research experience, sociodemographic characteristics of the study catchment area, facilities of recruitment for diarrhea case surveillance, and known care-seeking behavior for diarrheal illness. We also describe The Gambia's healthcare system and financing, current vaccine schedule and *Shigella* vaccine adaptation, local diarrhea management guidelines and challenges, and antibiotic resistance patterns in the region.

**Conclusions:**

The EFGH study in The Gambia will contribute to the multisite network of *Shigella* surveillance study and prepare the site for future vaccine trials. In addition, the data produced will inform policy makers about prevention strategies and upcoming *Shigella* vaccine studies among children in this setting.

## Teaser

This article describes The Gambia EFGH study site, research capacities, study catchment area, and recruitment facilities. This paper also highlights sociodemographic status, healthcare-seeking behaviors for diarrhea, diarrheal case management, antibiotic resistance patterns, and new vaccine adaptation in children.

**Figure 1.**
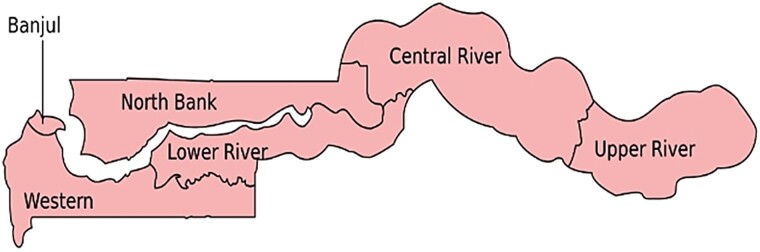
Administrative regions in The Gambia.

The Gambia is a small country in West Africa affectionately known as “the smiling coast of Africa,” covering approximately 11 000 km^2^, with 6 administrative regions (Figure 1) and a population of approximately 2.5 million people [1]. The median age is 17 years [2], life expectancy at birth is 62 years (World Bank, 2021, https://data.worldbank.org/country/GM), and infant mortality rate is 34.7 per 1000 live births [1]. The mean income per capita is US$771 per annum, less than half the sub-Saharan Africa average of US$1632, and 17.2% of the population live in poverty [1].

## MEDICAL RESEARCH COUNCIL UNIT THE GAMBIA AT THE LONDON SCHOOL OF HYGIENE AND TROPICAL MEDICINE

The Medical Research Council Unit The Gambia (MRCG), established in 1947 by the Medical Research Council United Kingdom (MRC UK), is one of the largest scientific research centers in sub-Saharan Africa. The MRCG became part of the London School of Hygiene and Tropical Medicine (LSHTM) in February 2018, according to the general strategy by the MRC UK to transfer its units to universities. The MRCG maintains its scientific independence within the LSHTM, and MRCG is considered an African-based academic research institution.

The MRCG at LSHTM has a large research portfolio of successful epidemiologic studies and clinical trials organized under 3 themes: disease control and elimination; vaccines and immunity; and nutrition and planetary health. These themes have been targeted to meet the health needs of low-income countries, reinforce regional and international collaborations, and address current priorities of the Sustainable Development Goals. MRCG at LSHTM has established strategic partnerships within the region through an alliance called the West African Global Health Alliance.

Over the past 75 years, MRCG at LSHTM has developed and maintained programs, infrastructure, and resources that support a wide spectrum of research activities, from basic science to the evaluation of interventions for the control of diseases of public health importance in sub-Saharan Africa. The unit's investigator-led research portfolio is strengthened by the availability of excellent laboratory facilities and easy access to the field, with a population willing to participate in health research. The unit provides excellent clinical services within its Clinical Services Department, maintains rigorous ethical procedures, and delivers Good Clinical Practice (GCP)–compliant clinical trials.

MRCG participated into 2 large diarrhea burden and etiology studies carried out between 2008 and 2018, namely, the Global Enterics Multicenter Study (GEMS) [3, 4] and the Vaccine Impact on Diarrhea in Africa (VIDA) study [5, 6]. Other research achievements include a large trial on the efficacy of *Haemophilus influenzae* type B vaccine [7] and the subsequent near-elimination of the disease, pioneering studies on the impact of insecticide-treated bed nets against malaria that supported their worldwide use [8, 9], studies demonstrating the impact of conjugate pneumococcal vaccines on pneumonia [10, 11] and child mortality, and the reduction of Hepatitis B carriage in The Gambia following vaccination [12]. A randomized controlled trial of enterotoxigenic *Escherichia coli* vaccine among children aged 6–18 months is ongoing to assess safety and efficacy on *E coli*–associated diarrhea, and has successfully completed enrollment of 4936 study participants [13].

## MRCG LABORATORY FACILITIES

The MRCG laboratory facility serves as a center of excellence for training in laboratory science within the West African region. Activities span from basic research in immunology, microbiology, virology, and molecular biology to large epidemiological studies and intervention trials as well as routine clinical diagnostics. Microbiology, clinical services, molecular biology, genomic, serology, and immunology are located at the main MRCG at LSHTM campus in Fajara. The Unit also supports 2 laboratories in its field stations in rural Gambia, in Basse and Keneba. Basse field station was established in 1982; has capacity for microbiology, molecular biology, and immunology; and supports all MRCG at LSHTM research activities in this region. The Basse laboratory was responsible for conventional pathogen detection, including *Shigella* culture, serotyping, and antimicrobial resistance, testing nearly 8700 stool samples collected from diarrhea cases and their matched controls in the GEMS and VIDA studies [3–5, 14].

## EFGH STUDY CATCHMENT AREA

The EFGH study site in The Gambia is based in Upper River Region (URR), in eastern Gambia (Figure 1), with a population of 230 000, and 1 major town called Basse. In URR, the temperature varies between 18°C and 44°C. There are distinctive dry (November–May) and wet (June–October) seasons, and the mean annual rainfall is 876 mm. The economy is primarily dependent on small-scale agriculture [15], and the main ethnic groups are the Mandinka, Fula, and Sarahulleh.

Malaria is endemic in URR, with the incidence of clinical malaria estimated between 0.08 and 1.89 per person-year during transmission season (July–December) [16]. Human immunodeficiency virus prevalence is low (<2%) [17]. Immunization coverage of all 3 doses of diphtheria, pertussis, and tetanus (DPT) was 81.7% [18]. The major drinking water sources in this region are boreholes, deep tube wells, and open wells, and some villages have a public water supply system piped to a communal tap that is shared by multiple households. Pit latrines are the most common type of toilet [19]. Only 10.2% of primary caregivers of children <5 years of age have attended primary and postprimary school [20], and the main mode of transport in rural villages are donkey carts and bicycles [15]. However, the use of commercial vehicles and motorbikes is increasing.

**Figure 2.**
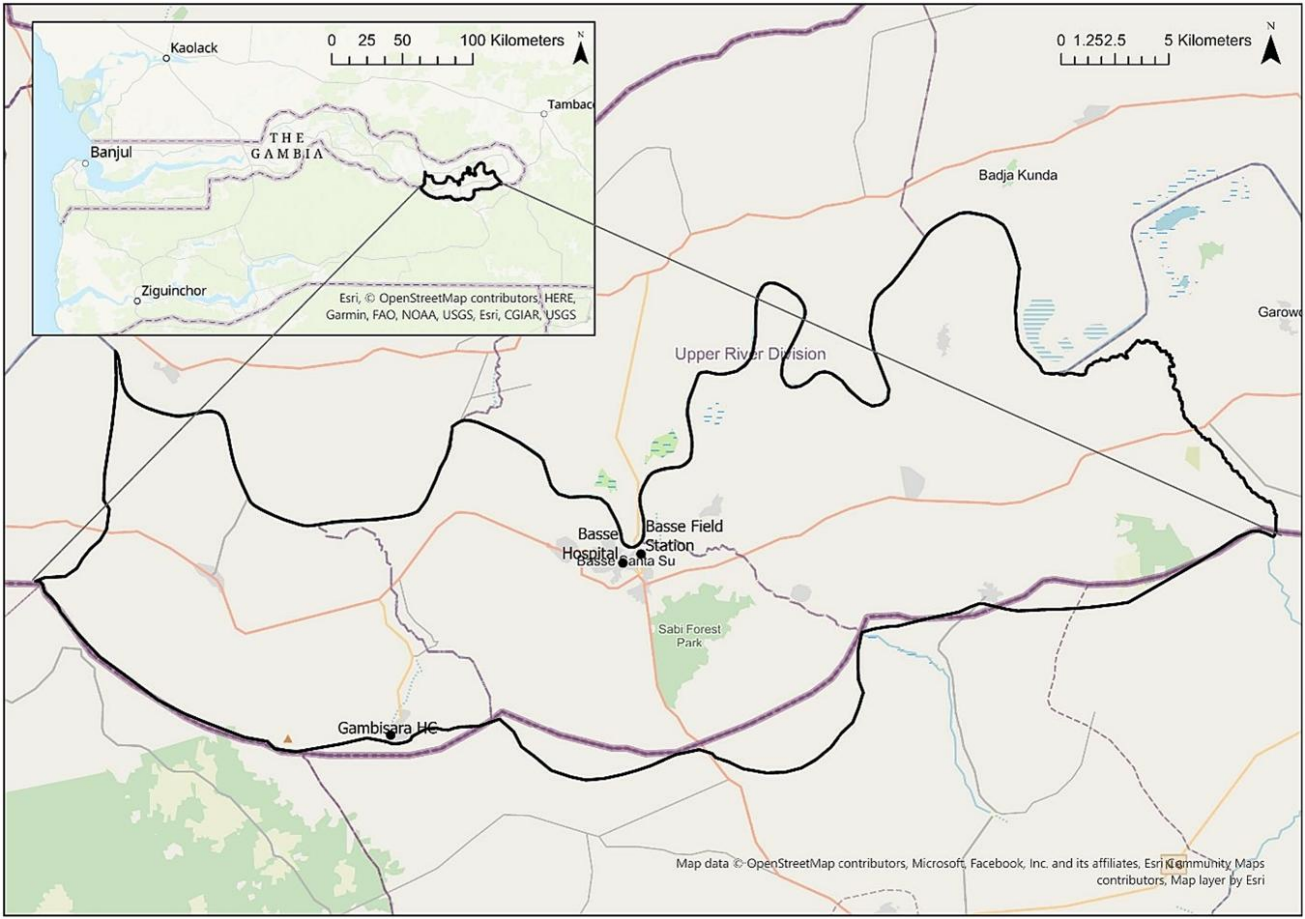
Enterics for Global Health Study catchment area map, The Gambia site.

The EFGH catchment area constitutes a subset of the population of the Basse Health and Demographic Surveillance System (BHDSS), which was established in 2007 and covers the south bank of the URR (Figure 2). The BHDSS includes 224 villages across 2 districts, with a population of 213 587, of whom 30 691 (14.4%) are children <5 years old (December 2022 MRCG Census data). The population is enumerated through household visits every 4 months, with records of births, deaths, vaccination, marriage, and migration in and out of the surveillance area. The Basse Field Station, which houses the microbiology laboratory and field office, is within the BHDSS area.

The EFGH catchment consists of 134 villages with a population of approximately 138 000. Within the EFGH catchment area there are 6 public health facilities, 1 district hospital (Basse), and 5 health centers/posts (Sabi, Demba Kunda, Gambisara, Sotuma, and Bakadagy). EFGH is recruiting from 2 of these facilities, Basse District Hospital and Gambisara Health Center, which were chosen because of the high enrollment in previous diarrhea studies, the large proportion of the total population served (65%), and proximity to MRCG Basse Field Station. Basse District Hospital is a referral hospital with an outpatient department, a general adult inpatient ward, pediatric ward, labor ward, minor theater, and a small laboratory. The hospital serves about 196 000 people in URR. Gambisara is a small rural health center that served as a GEMS and VIDA health facility and refers severe cases to Basse District Hospital. Both health facilities are open 24 hours a day and provide emergency services, adult medicine, and reproductive and child health services including free healthcare for children <5 years old.

## IMPLEMENTATION OF THE EFGH STUDY WITHIN THE GOVERNMENT HEALTH FACILITIES

The EFGH study staff integrate with the existing health facility staff to ensure acutely ill children are managed in accordance with The Gambian Ministry of Health guidelines [21] and the World Health Organization (WHO) Integrated Management of Childhood Illness guidelines [22]. The Ministry of Health guidelines are adapted from WHO guidelines. These guidelines are summarized in Table 1. In situations where guideline-recommended treatments are out of stock, the EFGH study supports treatment for study participants.

**Table 1.** Summary Table of Treatment Guidelines of Ministry of Health and Social Welfare, Republic of The Gambia

Dehydration Management		
**Children without severe acute malnutrition**		
Dehydration	Country guidelines (the same as WHO-indicated treatment)	
Severe (Plan C—facility)	Start IV fluid immediately (preferably Ringer’s lactate solution, 100 mL/kg). Reassess every 15–30 min. Give ORS (15 mL/kg/h) as soon as the child can drink. Reclassify dehydration after 6 h in infant and 3 h in child and continue with A, B, C plan.	Age <12 mo: 30 mL/kg^a^ in 1 h followed by 70 mL/kg over 5 h Age 12 mo–60 mo: 30 mL/kg^a^ in 30 min followed by 70 mL/kg over 2.5 h
Some (Plan B—facility)	Give recommended ORS in clinic over 4 hours. Reclassify dehydration after 4 h and continue with A, B, C plan.	Age* Up to 4 mo/weight <6 kg: 200–450 mL Age 4 mo up to 12 mo/weight 6 to <10 kg: 450–800 mL Age 12 mo up to 2 y/weight 10 to <12 kg: 800–960 mL Age 2 y up to 5 y/weight 12–19 kg: 960–1600 mL *Use the child's age only when you do not know the weight. The approximate amount of ORS required (in mL) can also be calculated by multiplying the child's weight (in kg) by 75.
None (Plan A—home)	Increase food and fluid intake to prevent dehydration	
**Children with severe acute malnutrition and no shock**		
Dehydration	Country guidelines (same as WHO-indicated treatment)	
Severe (Plan C—facility)	Give ReSoMal (or half-strength standard low ORS with added potassium and glucose) 5 mL/kg every 30 min for the first 2 h, and 5–10 mL/kg/h for the next 4–10 h on alternate hours with F75. If rehydration still required at 10 h, give starter F75 instead of ReSoMal, at the same times.	
Some (Plan B—facility)	Same as above	
None (Plan A—home)	Not applicable (because child admitted in health facility)	
**Therapeutic zinc**		
Population	Country guidelines (the same as WHO-indicated treatment)	
All children	Zinc supplementation for 10–14 d (age ≤6 mo: 10 mg/d; age >6 mo: 20 mg/d	
**Antibiotics**		
Population	Country guidelines (the same as WHO-indicated treatment)	
Dysentery	Ciprofloxacin (15 mg/kg) twice daily for 3 d OR based on local sensitivity. IV/IM ceftriaxone at 50–80 mg/kg/d for 3 d (if child is severely ill or as second-line treatment)	
Suspected cholera (age **≥**2 y + severe dehydration + cholera present in area)	Erythromycin (12 mg/kg) 4 times a day for 3 d Ciprofloxacin 10–20 mg/kg twice a day for 5 d Cotrimoxazole: 4 mg/kg trimethoprim and 20 mg/kg sulfamethoxazole twice a day	

Source: WHO [21, 22].

Abbreviations: IM, intramuscular; IV, intravenous; ORS, oral rehydration solution; ReSoMal, rehydration solution for malnutrition; WHO, World Health Organization.

^a^Repeat if the radial pulse is still very weak or not detectable.

## HEALTHCARE SYSTEMS AND FINANCING

The Gambia operates a 3-tier health system, namely primary, secondary, and tertiary healthcare levels. The primary level serves as the first point of contact for accessing healthcare in the community and provides health promotion, preventive, curative, and rehabilitative services. Nurses (community health, state-enrolled, and state-registered nurses) and public health officers are the main care providers in the primary level in community clinics, health posts, and minor health centers. The secondary healthcare level is composed of major health centers and regional hospitals, which receive referrals from the primary level. The tertiary healthcare level serves as the referral level for secondary facilities and provides specialized consultative healthcare for inpatients, advanced medical investigation, and patient management at teaching, general, and specialized hospitals [23]. Although there is no functional health insurance policy in place for the public healthcare system, healthcare service is free for children <5 years old and pregnant women (antenatal, childbirth, and postpartum period).

## CHALLENGES AFFECTING IMPLEMENTATION OF CLINICAL GUIDELINES AT HEALTH FACILITIES

Although The Gambia Ministry of Health has adopted the WHO diarrhea management guidelines, some practical implementation challenges are encountered. Health staff are not consistent in following the national and WHO guidelines, and antibiotics are frequently prescribed without the required indication [24]. The scarcity of recommended antibiotics, zinc supplements, and other drugs also hinders following recommended guidelines, and the available antibiotics are prescribed instead, even if not ideal. Low literacy and minimal formal health knowledge among caretakers of sick children can result in home instructions not being well-followed either [15].

## VACCINE SCHEDULE AND VACCINE ADAPTATION

The Gambian Expanded Programme on Immunization schedule for children under 5 (Table 2) currently follows the WHO recommendations and guidelines (Table 2). The Gambia has consistently documented complete DPT vaccination coverage >90%, but there has been lower coverage among poor and marginalized groups [25]. The government of The Gambia is supported by multilateral partners and in-country nongovernmental organizations to ensure that all children receive their basic vaccinations.

**Table 2.** Gambian Expanded Programme on Immunization Vaccination Schedule

Schedule	Vaccination			
At birth or soon after	BCG injection	Oral polio 0	Hepatitis B	…
2 mo	Pentavalent^a^ 1	Oral polio 1	PCV 1	Rota 1
3 mo	Pentavalent 2	Oral polio 2	PCV 2	Rota 2
4 mo	Pentavalent 3	Oral polio 3	PCV 3	…
9 mo	Measles/rubella	Yellow fever	Oral polio 4	…
12 mo	Meningitis A	DPT booster	…	…
18 mo	Measles/rubella	Oral polio 5	…	…

Abbreviations: DPT, diphtheria, pertussis, and tetanus; PCV, pneumococcal conjugate vaccine; Rota, rotavirus.

^a^Pentavalent, Diphtheria, pertussis, tetanus, hepatitis B, and *Hemophilus influenzae* b.

## *SHIGELLA* INCIDENCE, PREVALENCE, AND ANTIMICROBIAL RESISTANCE

In The Gambia, *Shigella* was the second leading cause of diarrhea identified in the earlier GEMS study, was one of the 5 most common pathogens responsible for diarrhea, and had highest incidence in older children in The Gambia compared to other GEMS sites in Africa and Asia, except Bangladesh [3, 4, 26]. In both GEMS and VIDA studies, the positivity of shigellosis was also higher in The Gambia compared to Mali and Kenya (Table 3, Table 4) [3, 4, 14, 26], and 30.8% of diarrhea cases were attributable to *Shigella* infection in The Gambia (Table 5) compared to 9.3% in Mali and 18.7% in Kenya [14]. In The Gambia, the incidence of *Shigella* is greatest during the rainy season, between June and September [14]. In the GEMS and VIDA studies, 10.6% and 12.9%, respectively, of moderate-to-severe diarrhea (MSD) cases were positive for *Shigella* by stool culture (Table 5). *Shigella flexneri* was the most common serogroup, followed by *Shigella boydii*, *Shigella sonnei*, and *Shigella dysenteriae* in both GEMS and VIDA studies (Table 6) [14].

**Table 3.** Attributable Fraction and Attributable Incidence of *Shigella* by Age Group in The Gambia Site for the Global Enterics Multicenter Study and Vaccine Impact on Diarrhea in Africa Study Based on Detection by Quantitative Polymerase Chain Reaction (TaqMan)

Age Group	VIDA (2015–2018)		GEMS (2008–2012)	
	AF (95% CI)	AI (95% CI)	AF (95% CI)	AI (95% CI)
0–11 mo	14.02 (9.3–17.1)	3.37 (2.3–4.1)	7.5 (3.9–12.9)	1.0 (.5–1.8)
12–23 mo	39.96 (36.5–46.2	8.83 (8.1–10.2)	32.7 (27.0–42.0)	6.5 (5.2–8.3)
24–59 mo	34.73 (28.9–40.2)	2.1 (1.7–2.4)	25.9 (18.5–36.1)	0.7 (.5–1.1)

Sources: Kotloff et al [3, 4].

Abbreviations: AF, attributable fraction; AI, attributable incidence per 100 child-years; CI, confidence interval; GEMS, Global Enterics Multicenter Study; VIDA, Vaccine Impact on Diarrhea in Africa.

**Table 4.** Proportion of Vaccine Impact on Diarrhea in Africa Study Participants From The Gambia in Each Age and Diarrhea Group With *Shigella* Detection Based on Detection by TaqMan Array Card (Cycle Threshold <35)

The Gambia	VIDA (2015–2018)			
	0–5 mo	6–11 mo	12–23 mo	24–59 mo
MSD				
Dysentery	6/20 (30.0%)	48/98 (49.0%)	170/205 (82.9%)	125/160 (78.1%)
Nondysentery	6/66 (9.1%)	79/355 (22.3%)	181/414 (43.7%)	155/360 (43.1%)
Controls	4/101 (4.0%)	78/595 (13.1%)	235/748 (31.4%)	190/694 (27.4%)

Source: Kasumba et al [14].

Proportion of *Shigella*/enteroinvasive *Escherichia coli* positive, where positive is defined as a cycle threshold <35.

Abbreviations: MSD, moderate-to-severe diarrhea that was medically attended; VIDA, Vaccine Impact on Diarrhea in Africa.

**Table 5.** Positivity of *Shigella* in Cases and Controls by Both Culture and Quantitative Polymerase Chain Reaction in the Vaccine Impact on Diarrhea in Africa Study, The Gambia (2015–2018)

Test	Cases, %	Controls, %
Stool culture	12.9	2.2
Positive (Ct <35)	45.9	30.2
Attributable to *Shigella*	30.8	

Source: Kasumba et al [14].

Abbreviation: Ct, cycle threshold.

**Table 6.** Distribution of *Shigella* Serogroup Among *Shigella* Culture-Positive Moderate-to-Severe Diarrhea Cases in the Global Enterics Multicenter Study and Vaccine Impact on Diarrhea in Africa Study, The Gambia

*Shigella* Serogroup	VIDA Study (2015–2018)	GEMS Study (2008–2012)
	Cases (n = 207), No. (%)	Cases (n = 116), No. (%)
*S flexneri*	146 (70.5)	80 (69.0)
*S boydii*	30 (14.5)	7 (6.0)
*S sonnei*	24 (11.6)	24 (20.7)
*S dysenteriae*	6 (2.9)	5 (4.3)

Source: Kasumba et al [14].

Abbreviations: GEMS, Global Enterics Multicenter Study; VIDA, Vaccine Impact on Diarrhea in Africa.

*Shigella-*attributable diarrhea appears to be increasing. Between 2008 and 2012, incidence in the GEMS study was estimated at 1.0 per 100 child-years in 0- to 11-month-olds, and 6.5 per 100 child-years in 12- to 23-month-olds [26]. Between 2015 and 2018, the incidence of *Shigella*-attributable MSD in the VIDA study was estimated at 3.6 (95% confidence interval [CI], 2.6–4.8) per 100-child-years in 0- to 11-month-olds and 9.8 (95% CI, 9.4–12.0) per 100 child-years in 12- to 23-month-olds (Kotloff et al, unpublished data).

In The Gambia, *Shigella* isolates were resistant to commonly used antibiotics. Almost all (95.0%) *Shigella* isolates were resistant to cotrimoxazole and half (48.4%) of them were resistant to ampicillin [14]. Resistance to the guideline-indicated first-line (ciprofloxacin), second-line (azithromycin), and third-line (ceftriaxone) antibiotics for *Shigella* was none or rare in 0.0%, 0.3%, and 0.3% of isolates, respectively [14]; these drugs are not commonly available in health facilities. The most commonly used antibiotics in The Gambia for MSD cases were ciprofloxacin (47.0%), metronidazole (29.3%), and co-trimoxazole (18.0%), and among dysenteric cases were ciprofloxacin (85.3%) and metronidazole (6.3%) [24].

## HEALTHCARE-SEEKING FOR DIARRHEA

Serial healthcare utilization surveys were conducted both in GEMS (2008–2012) and VIDA (2015–2018) studies in The Gambia. In GEMS, caretakers reported that 23.3% of the children experienced diarrhea within the previous 2 weeks. About 81%–85% of primary caregivers sought care outside the home for reported diarrhea in children <5 years old and 15% did not seek any care [15, 20]. Approximately half of the children with caregiver-reported diarrhea sought care in health centers [15, 20], while others sought care with private licensed practitioners (14.0%) or unlicensed practitioners (13.9%), bought remedies at the market (9.1%) or over the counter at a pharmacy (8.3%), or sought out traditional healers (4.8%) [15]. Reasons for not seeking formal healthcare included high cost of treatment, travel cost, lack of transport, distance to the health facility, more time needed to travel to health facility, and insufficient knowledge of danger signs among primary caretakers [15, 20].

## TRAINING AND CAPACITY BUILDING

The MRCG has diverse groups of researchers and staff from sub-Saharan Africa, including The Gambia and other West African countries, and from abroad (eg, Europe, Australia). MRCG at LSHTM maintains a strict portfolio of equity during employment and equal opportunities for all employees. Team leadership promotes a culture that values and respects equality, diversity, and inclusiveness where everyone feels comfortable sharing their perspective and ideas. Team members have equal access to the same resources and opportunities for career and professional development, including mentorship programs, networking opportunities, and funding for research projects.

The EFGH Gambia team comprises many young early career researchers, and actively promotes their independent research and career development. The EFGH junior investigators have participated in short courses offered by the University of Washington. Several junior investigators have also participated in EFGH supplement manuscript writing and submitted applications for the Rising Star Awards, a small fund which allows for nested studies within EFGH led by junior investigators from EFGH country sites. The EFGH study also facilitates distance learning for junior investigators to facilitate their career development.

## ETHICAL REVIEW

The EFGH study was approved by The Gambia government/Medical Research Council Unit, The Gambia Joint Ethics Committee, and the Institutional Review Board of the University of Maryland, Baltimore. Informed written consent was obtained from parents or guardians of study participants.

## CONCLUSIONS

The MRCG at LSHTM has excellent laboratory facilities at Fajara, Basse, and Keneba field stations, rigorous ethical procedures, and the ability to deliver GCP-compliant clinical trials. The MRCG at LSHTM has access to well-defined populations that are highly supportive of and willing to participate in its studies. The unit has excellent experience and willingness to participate in multicountry studies and to work in collaboration with other international research institutes. In the past, MRCG participated in 2 of the largest diarrhea burden and diarrhea etiology studies, GEMS and VIDA, which have provided an excellent data and infrastructure foundation, in addition to important historical data, for the current EFGH study. The EFGH Gambia site has a strong infrastructure, resources, and excellent trained staff to conduct diarrheal disease research. The *Shigella* burden is high and the EFGH study site can contribute substantially to future *Shigella* prevention studies and vaccine trials.

## Notes

***Acknowledgments.*** We express our deep gratitude to the families of the study participants in the EFGH studies; the clinical, data, field, and laboratory staff for their exceptional hard work and dedication; and the physicians, administration, and health officials at EFGH study sites who generously provided facilities and support for the conduct of the study. We are grateful to the EFGH Coordination team for their continuous extensive support to the study team whenever it is needed, including training and coordination of activities. Our special thanks to the GEMS and VIDA teams.

***Financial support.*** This research was supported by the Bill & Melinda Gates Foundation (grant numbers INV-016650, INV-031791, INV-028721, INV-041730) and by the UK Research and Innovation Medical Research Council (program number MC_UU_00031/1—Disease Control and Elimination).

***Supplement sponsorship.*** This article appears as part of the supplement “Enterics for Global Health (EFGH) *Shigella* Surveillance Study-Rationale and Methods,” sponsored by the Bill & Melinda Gates Foundation.
